# Neuronal subclass-selective proteomic analysis in *Caenorhabditis elegans*

**DOI:** 10.1038/s41598-020-70692-w

**Published:** 2020-08-13

**Authors:** Shunsuke Aburaya, Yuji Yamauchi, Takashi Hashimoto, Hiroyoshi Minakuchi, Wataru Aoki, Mitsuyoshi Ueda

**Affiliations:** 1 Division of Applied Life Sciences, Graduate School of Agriculture, Kyoto University, Kitashirakawa Oiwake-cho, Sakyo-ku, Kyoto, 606-8502 Japan; 2 Japan Society for the Promotion of Science, Kitashirakawa Oiwake-cho, Sakyo-ku, Kyoto, 606-8502 Japan; 3 Kyoto Monotech, 1095 Shuzei-cho, Kamigyo-ku, Kyoto, 602-8155 Japan; 4 JST, Precursory Research for Embryonic Science and Technology (PREST), 7 Goban-cho, Chiyoda-ku, Tokyo, 102-0076 Japan; 5 JST, Core Research for Evolutionary Science and Technology (CREST), 7 Goban-cho, Chiyoda-ku, Tokyo, 102-0076 Japan; 6 Kyoto Integrated Science and Technology Bio-Analysis Center, 134 Chudoji Minamimachi, Simogyo-ku, Kyoto, 600-8813 Japan

**Keywords:** Proteomics, Molecular neuroscience

## Abstract

Neurons are categorised into many subclasses, and each subclass displays different morphology, expression patterns, connectivity and function. Changes in protein synthesis are critical for neuronal function. Therefore, analysing protein expression patterns in individual neuronal subclass will elucidate molecular mechanisms for memory and other functions. In this study, we used neuronal subclass-selective proteomic analysis with cell-selective bio-orthogonal non-canonical amino acid tagging. We selected *Caenorhabditis elegans* as a model organism because it shows diverse neuronal functions and simple neural circuitry. We performed proteomic analysis of all neurons or AFD subclass neurons that regulate thermotaxis in *C. elegans*. Mutant phenylalanyl tRNA synthetase (MuPheRS) was selectively expressed in all neurons or AFD subclass neurons, and azido-phenylalanine was incorporated into proteins in cells of interest. Azide-labelled proteins were enriched and proteomic analysis was performed. We identified 4,412 and 1,834 proteins from strains producing MuPheRS in all neurons and AFD subclass neurons, respectively. *F23B2.10* (RING-type domain-containing protein) was identified only in neuronal cell-enriched proteomic analysis. We expressed *GFP* under the control of the 5′ regulatory region of *F23B2.10* and found *GFP* expression in neurons. We expect that more single-neuron specific proteomic data will clarify how protein composition and abundance affect characteristics of neuronal subclasses.

## Introduction

Neurons are categorised into several neuronal subclasses, each with different morphology, expression patterns, connectivity and function^1^. For example, in feeding *Drosophila*, two neurons, called feeding neurons, regulate sugar-induced feeding behaviour^2^ and in *Caenorhabditis elegans,* AFD (amphid finger cell D) neurons regulate thermotaxis^3–5^. Understanding molecular mechanisms of regulation of these neurons requires analysis of molecular details for each neuronal subclass^6,7^.

Single-cell RNA-seq analysis is used to assess expression patterns in neurons of mice^8,9^, *Drosophila*^10^ and *C. elegans*^11^ and new neuronal subclasses are now recognised. However, mRNA transcript and protein abundance do not correlate^12,13^. Further post-translational modifications, such as phosphorylation and ubiquitination are important for regulating cell functions^14,15^. Therefore, analysis of abundance and expression patterns of proteins in target subclass neurons is needed to identify molecular mechanisms underlying neuronal functions. Fluorescent reporters are commonly used to assess protein expression, but observation of individual expression patterns of proteins is difficult in a highly multiplex manner using this method^16^.

Comprehensive protein expression analysis enables identification of unique proteins in target subclasses and molecular mechanisms underlying neuronal functions^6,7^. Protein expression patterns of some subclasses were generated with mass spectrometry-based proteomic analysis^2,5,17^. In these studies, in vitro differentiation^8^, laser dissection^18,19^, flow cytometric sorting^20^, and antibody-coupled microbeads^21^ were used to isolate specific neuronal subclasses or all neuronal cells. However, these methods have some limitations. For example, in vitro differentiated neurons do not form neuronal networks like brains. Laser dissection is challenging to use for isolating branched and interconnected cells. Flow cytometric sorting and antibody-coupled microbead methods require long sample preparation times to dissociate cells^21^.

In vivo cell-selective metabolic labelling of proteomes solve these problems and generates a comprehensive proteomic analysis for targeted cells^22–25^. These methods fall into two groups. One is protein biotinylation, involving expression of an engineered ascorbate peroxidase or engineered *Escherichia coli* biotin ligase in target cells^22,25^. The second is protein azidation involving expression of a mutant aminoacyl-tRNA synthetase in target cells. The method is termed cell-selective bio-orthogonal non-canonical amino acid tagging (BONCAT)^23,26,27^. As the name implies, biotinylation entails labelling proteins in target cells with biotin and recovering biotinylated proteins using biotin-streptavidin interaction. Unfortunately, biotinylated proteins display toxicity for target cells^25^. Protein azidation entails labelling newly synthesised proteins with azide-containing amino acids and recovering these proteins with copper-catalysed azide-alkyne cycloaddition. Azide labelled proteins are known to display low toxicity toward living cells^26^.

To date, proteomic analysis of neurons using in vivo cell-selective labelling is only reported in mice and *Drosophila*^27,28^. BONCAT approaches have not been applied much in *C. elegans*. Proteomic analysis using in vivo cell-selective labelling is reported only in pharyngeal muscle^23^. Another example is proteomic analysis of newly synthesized proteins using a protein azidation method^29^.

In this study, we first used a neuronal subclass-selective proteomic analysis with cell-selective BONCAT using monolithic nano LC–MS/MS. We found 4,412 proteins from all neurons and 1,834 proteins from AFD subclass neurons, with low background. Identified proteins were uniquely expressed in neurons, and a GFP-reporter assay confirmed their expression profiles. The feasibility of neuronal subclass-selective proteomic analysis in *C. elegans* is now demonstrated, and we can expect additional single-neuron specific proteomic data to clarify how protein composition and abundance affect characteristics of each neuronal subclass.

## Materials and methods

### Worm maintenance

*Caenorhabditis elegans* N2 (Bristol) strain and *Escherichia coli* OP50-1 strain (*ura*^*−*^, *strR*) were obtained from the *Caenorhabditis* Genetics Center (CGC). Worms were cultured and maintained on nematode growth medium (NGM) plates with *Escherichia coli* OP50-1 in 3 cm or 6 cm dishes^30^.

### Plasmids used in this study

pGH8 was a gift from Erik Jorgensen (Addgene plasmid # 19359; https://n2t.net/addgene:19359; RRID: Addgene_19359)^31^. pKPY197 was a gift from David Tirrell (Addgene plasmid # 62599; https://n2t.net/addgene:62599; RRID: Addgene_62599)^23^. pCFJ104 (Pmyo-3::mCherry::unc-54 3′UTR*)* was a gift from Erik Jorgensen (Addgene plasmid # 19328; https://n2t.net/addgene:19328; RRID: Addgene_19328)^31^. pKPY514 was a gift from David Tirrell (Addgene plasmid # 62598; https://n2t.net/addgene:62598; RRID: Addgene_62598)^23^. pHW394 (15xUAS::GFP::let-858 3′UTR) was a gift from Paul Sternberg (Addgene plasmid # 85584 ; https://n2t.net/addgene:85584; RRID:Addgene_85584)^32^. pF25B3.3p::mcherry was constructed previously^33^.

To construct pKPY197-Prab3 and pKPY197-Pgcy-8, pKPY197^23^ was digested with *Sal*I. The *rab-3* promoter fragment and *gcy-8* promoter fragment were cloned from pGH8 and *C. elegans* genome, respectively. Each fragment was inserted into the digested pKPY197 plasmid. Primers used in this study are provided in Supplementary Table 1.

To construct pF23B2.10p::GFP, the 5′ regulatory region of F23B2.10 was cloned from the *C. elegans* genome and inserted into pCFJ90. pCFJ90 with the 5′ regulatory region of F23B2.10 was linearised without the mCherry region by PCR amplification. *GFP* (S65C) were amplified from pHW394 and these fragments were joined.

### Construction of transgenic strains

Injections were performed into an N2 background with the aid of a stereomicroscope (SZX10; Olympus, Tokyo, Japan) equipped with a Femtojet 4i (5,252 000.021; Eppendorf, Hamburg, Germany) and Femtotips II (1,501,040; Eppendorf). The strain SA1 (*SAIs1[Prab-3::frs-1(Thr412Gly)::fib-1/rps-16::gfp(S65C, synIVS)::unc-54 3′UTR]*) was generated by co-injecting two plasmids [10 ng/µL of pKPY197-Prab3 and 90 ng/µL of pUC19 in water] into *C. elegans*. Extrachromosomal arrays of three GFP-expressing lines were integrated into the *C. elegans* genome by UV irradiation^34^. Strain SA2 (*SAIs2[Pgcy-8::frs-1(Thr412Gly)::fib-1/rps-16::gfp(S65C, synIVS)::unc-54 3′UTR, Pmyo-3::mcherry]*) was generated by co-injecting two plasmids [45 ng/µL of pKPY197-Pgcy-8 and 5 ng/µL of pCFJ104 in water] into *C. elegans*. Extrachromosomal arrays of two GFP-expressing lines were integrated into *C. elegans* genome by UV irradiation. The strain SA3 (*SAEx3[Pf23b2.10::GFP::unc-54 3′UTR, Pf25b3.3::mcherry]*) was generated by co-injecting two plasmids [35 ng/µL of pF23B2.10p::GFP, 50 ng/µL of pF25B3.3p::mcherry and 15 ng/µL of pUC19 in water] into *C. elegans*.

### Labelling *C. elegans* with azido-phenylalanine

*Escherichia coli* KY33 [pKPY514], a gift from David Tirrell^23^, is an arginine-, lysine- and phenylalanine-auxotrophic strain^23^. *Escherichia coli* KY33 was labelled with azido-phenylalanine following methods in a previous report^23^. Worms were precultured with 5 mL of S medium^30^ supplemented with 15 mg/mL *E. coli* KY33 cultured with phenylalanine at 20 °C, 250 rpm. Precultured worms were pelleted by centrifugation at 300 g for 5 min at room temperature and washed with 1 mL of S medium. This procedure was repeated three times. The *C. elegans* pellet was suspended in 5 mL of S medium supplemented with 15 mg/mL *E. coli* KY33 cultured with azido-phenylalanine and incubated at 20 °C and 250 rpm for 24 h.

Labelled nematodes were recovered using a 20 µm nylon filter (pluriStrainer 20 µm; pluriSelect, Leipzig, Germany) and washed with 5 mL of M9 buffer (0.6% w/v Na_2_HPO_4_ (Nacalai Tesque, Kyoto, Japan), 0.3% KH_2_PO_4_ (Nacalai Tesque), 0.5% NaCl (Nacalai Tesque)). Nematodes were recovered by centrifugation at 300 g for 5 min and processed in subsequent procedures.

### Fixation of nematodes and TAMRA staining

Nematodes were fixed and labelled with dibenzocyclooctyne-PEG4-Fluor 545 (TAMRA-DBCO; Sigma-Aldrich, St. Louis, MO, USA) as described in a previous report^23^.

### Fluorescence microscopy

A 2% agarose pad was prepared, onto which 5 µL of 1 mM levamisole (Tokyo Chemical Industry Co., Ltd., Tokyo, Japan) in M9 buffer was placed. Worms were picked up and placed onto the agarose pad with levamisole, over which a cover glass was gently placed. Fluorescence was observed by confocal laser scanning microscopy (LSM700; Carl Zeiss, Oberkochen, Germany). Fluorescence of GFP, TAMRA and mCherry were observed using 488 nm, 555 nm and 561 nm lasers, respectively. Acquired images were processed using Zen Lite and ImageJ^35^.

### Sample preparation for neuronal subclass-selective proteomics

Lysis buffer (8 M Urea (Nacalai Tesque), 4% CHAPS (Dojindo, Kumamoto, Japan) and 1 M NaCl, in 200 mM Tris-HCl pH 8.0 (Nacalai Tesque)) was added to the labelled worms and the solution was sonicated on ice using a probe sonicator (Q125, Q Sonica; Newtown, CT, USA) equipped with an 1/8″ probe (10 s on, 10 s off, 1,000 J). Sonicated samples were centrifuged for 5 min at 10,000 g. We selectively enriched azide phenylalanine (Azf)-labelled proteins using a Click-iT™ Protein Enrichment Kit, for click chemistry capture of azide-modified proteins (Thermo Fisher Scientific, Waltham, MA, USA) following the manufacture's protocol. The enriched samples were digested with trypsin on an alkyne-agarose column. Digested samples were desalted with MonoSpinC18 (GL sciences, Osaka, Japan) and dried by vacuum centrifugation. Dried peptides were dissolved in 25 µL of 0.1% formic acid (Wako, Osaka, Japan).

### Nano LC–MS/MS analysis

Proteomic analysis was conducted as described in a previous report^36^. Briefly, 5 µl samples were injected, and peptides were separated using a liquid chromatography (LC, Ultimate 3,000; Thermo Fisher Scientific)—tandem mass spectrometry (MS/MS, LTQ Orbitrap Velos Mass Spectrometer; Thermo Fisher Scientific) system equipped with a long monolithic silica capillary column (490 cm , 75 μm internal diameter) at a flow rate of 280 nL/min. A gradient was achieved by changing the ratio of two eluents: eluent A, 0.1% (v/v) formic acid and eluent B, 80% acetonitrile containing 0.1% (v/v) formic acid. The gradient began with 5% B, increased to 45% B for 750 min, further increased to 95% B to wash the column for 140 min, then returned to the initial condition and held for re-equilibration. The separated analytes were detected using a mass spectrometer with a full scan range of 350–1,500 m/z (resolution, 60,000), followed by ten data-dependent collision-induced dissociation MS/MS scans. The temperature of the ion transfer tube was set to 280 °C, and dynamic exclusion was 180 s. Electrospray ionisation voltage was set at 2.3 kV.

### Data analysis

Data analysis was performed using Proteome Discoverer 2.1 (Thermo Fisher Scientific). Protein identification was performed using MASCOT (Matrix Science, London, UK) against the *C. elegans* UniProt protein database (27/08/17) with a precursor mass tolerance of 20 ppm and a fragment ion mass tolerance of 0.8 Da. Carbamidomethylation of cysteine was set as a fixed modification and oxidation of methionine and acetylation of protein N-terminals were set as dynamic modifications. Data were filtered using a cutoff criteria of ≤ 0.01 (*q* value), corresponding to a 1% false discovery rate on a spectrum level. Tissue enrichment analysis (TEA) was performed on WormBase.org^37,38^.

## Results

### Experimental scheme

**Figure 1:**
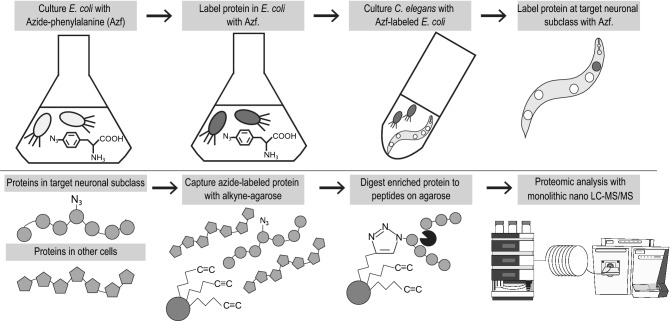
Experimental scheme of cell-selective BONCAT. *Escherichia coli* was cultured with azide-phenylalanine (Azf) to label proteins in *E. coli* with Azf. Next, *C. elegans* was cultured with the Azf-labelled *E. coli* to label proteins at target subclass neurons with Azf. After the extraction of total protein, azide-modified proteins in target subclass neurons were enriched using alkyne agarose. The enriched proteins were digested with trypsin and proteomic analysis was conducted with monolithic nano LC–MS/MS.

Neuronal subclass-specific proteomic analysis is shown in Fig. 1. We constructed transgenic *C. elegans* strains that produce MuPheRS and GFP in target neuronal subclass. MuPheRS replaces the Thr412 of PheRS to Gly. This substitution makes it possible to incorporate Azf more efficiently. Therefore, it is possible to label MuPheRS-expressing cells with Azf^23^. The transgenic *C. elegans* strains are cultured with Azf-labelled *E. coli* to label proteins at the target neuronal subclass with Azf. After enrichment of the azide-labelled proteins using alkyne-agarose, we can conduct proteomic analysis with monolithic nano LC–MS/MS to reveal proteomic composition for cells of interest (Fig. 1).

### Strain construction

**Figure 2:**
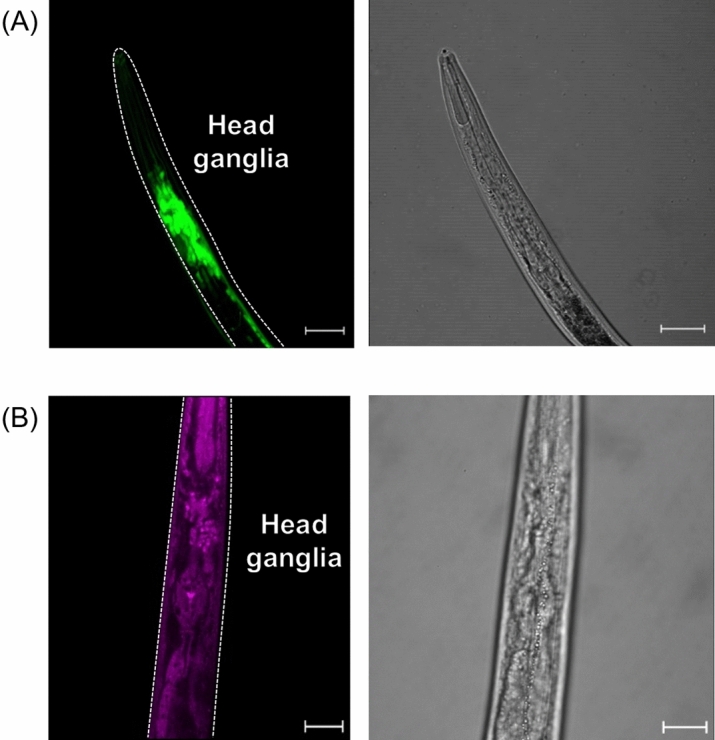
Confirmation of MuPheRS production and activity in the SA1 strain. (**A**) Confirmation of MuPheRS production in targeted cells in the SA1 strain (*SAIs1[Prab-3::frs1(Thr412Gly)::fib-1/rps-16::gfp(S65C, synIVS)::unc-54 3′-UTR]*). Green fluorescent protein was produced under the *rab-3* promoter (All neurons). A dotted white line indicates body shape. Scale bar indicates 20 μm. (**B**) Confirmation of azide-phenylalanine incorporation in targeted cells. Azide-proteins were stained with dibenzocyclooctyne-PEG4-Fluor 545 (TAMRA-DBCO). Scale bar indicates 20 μm. The SA1 strain was cultured with Azf-labelled *E. coli* KY33 and stained with TAMRA-DBCO.

We generated transgenic *C. elegans* strains that produce MuPheRS and GFP under the *rab-3* promoter (SA1 strain (*SAIs1[Prab-3::frs1(Thr412Gly)::fib-1/rps-16::gfp(S65C, synIVS)::unc-54]*))^39^ or *gcy-8* promoter (SA2 strain (*SAIs2[Pgcy-8::frs1(Thr412Gly)::fib-1/rps-16::gfp(S65C, synIVS)::unc-54, Pmyo-3::mcherry]*))^40,41^. It was known that the *rab-3* promoter drives expression in all neurons^39^, and *gcy-8* promoter in AFD neurons^40,41^. AFD neurons are the thermosensory neurons essential for thermotaxis^42^. We chose AFD neurons as a model for production of MuPheRS in a single neuronal subclass^41^. We observed the SA1 strain by fluorescence microscopy, and confirmed GFP fluorescence in targeted cells (Fig. 2A). Next, the SA1 strain was fixed with TAMRA-DBCO to visualise Azf incorporation. As a result, we successfully detected TAMRA fluorescence in neurons only when the SA1 strain was cultured with Azf-labelled *E. coli* KY33 and stained with TAMRA-DBCO (Fig. 2B, Supplementary Fig. 1A). In the SA1 strain cultured with Azf-labelled *E.coli* KY33 and not stained with TAMRA-DBCO, we did not observe strong TAMRA fluorescence signals (Supplementary Fig. 1A (iii)). However, high background fluorescence signals were observed in the whole body in both wild type N2 strain cultured with Azf- or Phe-labelled *E. coli* KY33 and stained with TAMRA-DBCO (Supplementary Fig. 1A (i), (ii)). We also successfully detected TAMRA fluorescence in AFD neurons using the SA2 strain (Supplementary Fig. 1B). The TAMRA fluorescence was observed around the anatomical position of AFD neurons. Furthermore, we did not observe strong TAMRA fluorescence signals in other cells. These results confirmed that the constructed strains produced MuPheRS and incorporated Azf in the targeted cells.

### All neuron-selective proteomics

**Figure 3:**
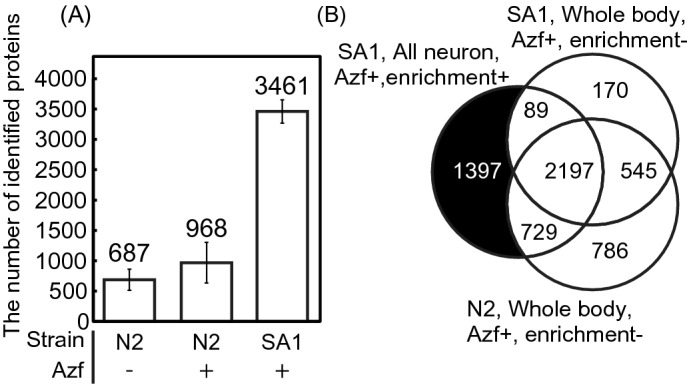
Comparison of the number of identified proteins. (**A**) The average number of identified proteins of Phe-cultured N2, Azf-cultured N2, and Azf-cultured SA1 with azide-protein enrichment. The data are shown as mean ± standard errors of the mean (N = 3). (**B**) Venn diagram to compare the compositions of proteins identified in worms fed on KY33 strain cultured with azide-phenylalanine. The numbers in the venn diagram indicate the number of proteins identified at least once in each sample (N2 or SA1). We performed tissue enrichment analysis with the black filled section (proteins identified only in SA1 strain cultured in the Azf-labeled KY33 strain with azide-protein enrichment procedure, Supplementary Table 3).

Proteomic analysis with the SA1 strain demonstrated the molecular composition of neurons. Non-specific incorporation of Azf into cells and non-specific adsorption to agarose was assessed from proteomic analyses using the N2 strain cultured with Azf- or Phe-labelled *E. coli* KY33 followed by enrichment of azide-labelled proteins with alkyne-agarose as control experiments of proteomic analysis. Proteomic analysis was conducted with monolithic nano LC–MS/MS^36^. We identified 3,461 proteins in the Azf-labelled SA1 strain (average of three biological replicates). The number of proteins identified in N2 strain cultured with the Phe-labelled *E. coli* KY33 was 687 proteins and that in the N2 strain cultured with the Azf-labelled KY33 was 968 proteins (Fig. 3A and Supplementary Table 2). We estimated that the number of proteins identified by non-specific adsorption to agarose was about 687 and the number of proteins identified by Azf-incorporation into non-specific cells was about 281 proteins. Little Azf-incorporation into non-specific cells was observed with cell-selective BONCAT. This analysis also supports that the TAMRA fluorescence observed in the control experiments (Supplementary Fig. 1A) was not derived from non-specific incorporation of Azf but non-specific adsorption of TAMRA-DBCO.

**Figure 4:**
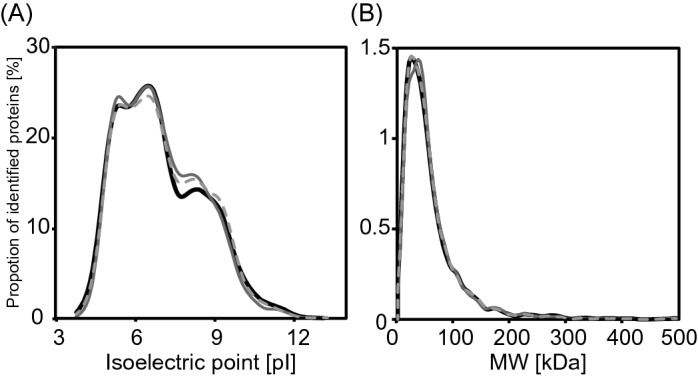
Distribution of the identified proteins in (**A**) isoelectric point and (**B**) molecular weight. The lines indicate the distribution of isoelectric point and molecular weight of the identified proteins. The black line shows the data of N2 strain cultured with the azide-labelled *E. coli* KY33 without azide-protein enrichment. The light grey dashed line and the dark grey line show the data of the SA1 strain cultured with the azide-labelled KY33 without or with azide-protein enrichment, respectively.

Next, we prepared proteomic samples of N2 and the SA1 strain cultured with the Azf-labelled *E. coli* KY33 without azide-labelled protein enrichment and prepared proteomic analyses (unenriched N2 and SA1). We compared compositions of proteins to the SA1 strain cultured in the Azf-labelled KY33 with azide-protein enrichment (enriched SA1) and to composition of unenriched N2 and SA1 (Fig. 3B and Supplementary Table 2). To confirm the presence of nonspecific adsorption during the enrichment process, the number of identified proteins was compared for two parameters obtained from protein sequence information; molecular weight and isoelectric point. We did not find detection bias between enriched and unenriched proteomic analysis (Fig. 4). We did identify 1,397 proteins only in the enriched SA1 proteomic analysis. These proteins included G protein-coupled receptors (GPCRs; e.g. 12 neurotransmitter receptors, including SRX-29, SRU-6) and representative neuronal proteins, such as RAB-3 (Ras-related protein, Rab-3)^39^. To validate the quality of the neuronal protein identification in SA1 strains, we conducted tissue enrichment analysis^37^, and annotated localisation of identified proteins was assessed with proteins identified only in the Azf-labelled SA1 strain (Fig. 3B, the black filled section). Enrichment of Posterior ventral neuron D (PVD), Inner labial neuron 2 (IL2), Side dorsal neuron derived from QL (SDQL), Amphid wing B cells (AWB), Amphid interneurons Y (AIY), Cephalic male neuron (CEM), outer labial sensillum, lateral ganglion and pharyngeal interneuron proteins was observed in proteins identified only in the Azf-labelled SA1 strain (Supplementary Table 3). Furthermore, among the proteins identified only in the enriched proteomic analysis, 1,004 proteins were known to express in neurons (Supplementary Table 2)^38^. However, we did not observe enrichment for neurons in identified proteins of unenriched N2 and SA1 (Fig. 3B, the grey filled section). These results strongly suggest that our analysis enriched neuronal proteomes and successfully identified neuron-specific proteins.

### Localisation analysis of the newly identified neuron-specific proteins

**Figure 5:**
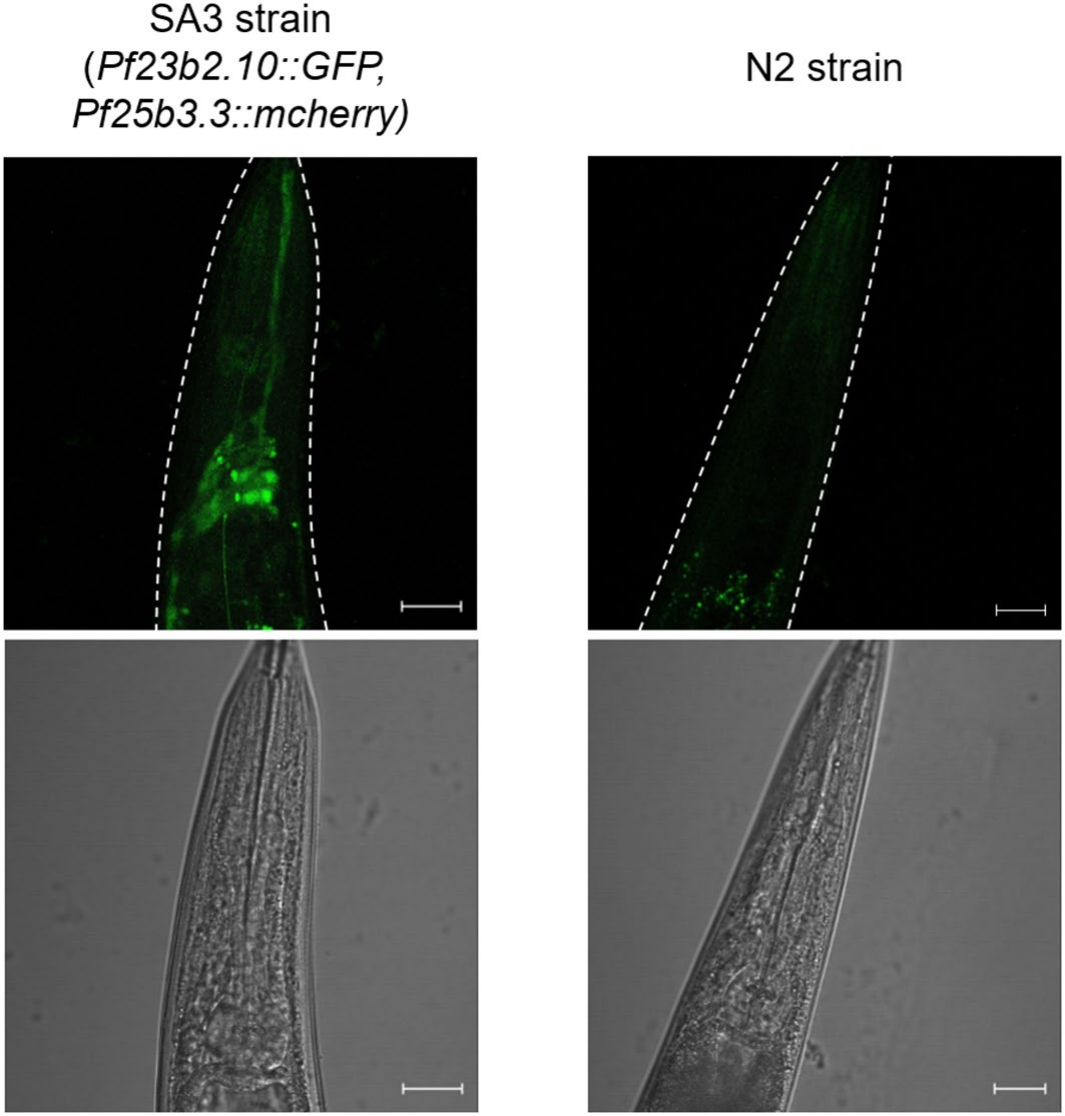
*GFP* expression under the control of the 5′ regulatory region of *F23B2.10.* Confocal imaging of the head ganglia of the SA3 strain (*SAEx3[Pf23b2.10::GFP, Pf25b3.3::mcherry]*) and N2 strain. We detected GFP fluorescence at some neurons only in the SA3 strain. Scale bar indicates 20 μm.

To verify our analysis, we searched for neuron-specific proteins identified in this study that lacked any expression pattern description in WormBase^38^ among neuron-specific proteins identified in this study. We identified several such proteins (Supplementary Table 4). We selected *F23B2.10* and cloned the 5′ regulatory region of this gene from the *C. elegans* genome and we successfully constructed the SA3 strain (*SAEx3[Pf23b2.10::GFP, Pf25b3.3::mcherry]*). In this strain, we verified the *Pf23b2.10*-drived production of GFP in neurons (Fig. 5).

### AFD subclass neuron-selective proteomic analysis using the *gcy-8* promoter

We specifically produced MuPheRS in AFD subclass neurons using the *gcy-8* promoter to carry out single neuronal subclass-selective proteomic analysis. The molecular mechanism for thermosensation is not fully clarified^3,4,41,42^. Previously, Kobayashi et al.^5^ carried out phosphoproteomic analysis of in vitro differentiated AFD subclass neuron. However, the protein composition of these cells is not established. Protein composition in AFD neuronal cells might provide some insights into the molecular mechanisms under thermosensation in *C. elegans*^42^. The SA2 strain was cultured with Azf-labelled KY33 and Azf-labelled protein was subjected to proteomic analysis.

**Figure 6:**
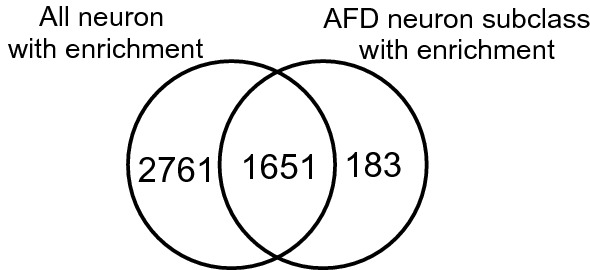
Venn diagram for comparison of proteome between the azide-enriched SA1 and SA2 strains cultured with the Azf-labelled KY33. Numbers in the Venn diagram indicate the number of identified proteins at least once in each fraction.

We found 1,834 proteins with the enriched proteomic analysis (Supplementary Table 2), and identified TAX-6, which is known to be produced in AFD^43^. We compared the proteomic analysis of the SA2 strain with the analysis from the SA1 strain cultured with Azf-labelled *E. coli* KY33 (Fig. 6). This comparison yielded 183 proteins only present in the AFD neuron-enriched proteomic analysis (Supplementary Table 2). Furthermore, among the 1,834 proteins identified in the SA2 strains in our proteomic analysis, 1,143 proteins were also identified in single-cell transcriptomic analysis of AFD neurons (Supplementary Table 2)^44^. Considering that 687 proteins were identified as non-specific adsorption of proteins to alkyne-agarose (Fig. 3), the match between single-cell transcriptomic analysis and our proteomic analysis indicated that we successfully enriched proteins produced in AFD subclass neurons.

## Discussion

We report, for the first time, proteomic analyses against all neurons and AFD subclass neurons using cell-selective BONCAT, and identify 4,412 proteins and 1,834 proteins, respectively (Figs. 3, 6). In the proteomic analysis against whole body, we identified 4,257 proteins, equivalent to the number identified in all neurons (Fig. 3B). Proteome coverage from all neuronal cell-selective BONCAT is as high as that of the whole-body proteome. This proteome is sufficient for searching out new marker proteins and elucidating molecular dynamics. Identified proteins from all neurons or AFD subclass neurons, neuron marker proteins, such as RAB-3^39^ or TAX-6^43^, were identified along with GPCRs and neuropeptides (Supplementary Table 2). The result of tissue enrichment analysis validated the quality of neuronal protein identification (Supplementary Table 2). Further, we verified localisation of a newly identified protein encoded by *F23B2.10* in neurons (Fig. 5). These results indicate that we can successfully enrich proteins by cell-selective BONCAT. Further analysis of identified proteins, including GPCRs, will provide deeper insights into neuronal functions. We also successfully performed a single neuronal subclass-selective proteomic analysis using about 100,000 worms cultured in 5 mL of S medium. Thus, we can extend the cell-selective BONCAT approach to any neuronal subclass even if it contains only a single neuron. We did not use quantitative proteomic approaches, such as SILAC, in this study. In further studies, quantitative proteomic data will be necessary to identify proteomic variances in neuronal cells of interest. Besides, it will be necessary to use several promoters for identification of the confident set of proteins that are specifically expressed in neuronal cells of interest. In this study, we identified 1,397 proteins only in the enriched SA1 proteomic analysis (Fig. 3). Among them, the localization of 43 proteins has not revealed (Supplementary Table 4), and we performed GFP reporter assay with *F23B2.10*, whose localization and function are unknown. As a result, we verified the *Pf23b2.10*-drived production of GFP in neurons (Fig. 5). GFP reporter assay is the best method for validating the results of the neuronal subclass selective proteomics analysis, but we only performed GFP reporter assay only *F23B2.10*. Therefore, we need to perform more reporter assays to confirm the localization of other proteins identified only in SA1 and SA2 strains. In a further study, we should verify the localization and function of unique proteins, and these insights will lead to the understanding of molecular mechanisms underlying the functions of neuronal cells and AFD neurons.

In our result, TAMRA fluorescence was also observed when N2 worms were cultured with Azf-labeled *E. coli* KY33 and stained with TAMRA-DBCO (Supplementary Fig. 1A (i)). However, the fluorescence was also observed in wild type N2 cultured with Phe-labeled *E. coli*. These results suggested that the fluorescence observed in the control experiments was not due to Azf taken up by non-target cells, but rather non-specific adsorption of TAMRA-DBCO. The results in our proteomic analyses of the negative controls support the non-specific adsorption of TAMRA-DBCO (Fig. 3). From the proteomic study of the negative control, 281 proteins were identified as non-specific incorporation of Azf into other cells and 687 proteins were identified as non-specific binding of proteins to alkyne-agarose (Fig. 3). To reduce the background fluorescence in the control experiments, we need to optimize the wash protocol or concentration of TAMRA-DBCO.

In the SA2 strains, fluorescence signals derived from TAMRA-DBCO were weak (Supplementary Fig. 1B). However, the position of the fluorescence signal was around the anatomical position of AFD neurons. Furthermore, proteomic analysis of the SA2 strain cultured with Azf-labeled *E. coli* also supports that we successfully enriched proteins derived from AFD neurons (Supplementary Table 2). Considering these points, we concluded that the cells strained with TAMRA-DBCO in the SA2 strain were AFD neurons.

In this study, we could not identify all neuronal and AFD neuronal marker proteins present in trace amounts using our proteomic analysis. These markers include GCY-8, GCY-18, GCY-27, FLP-6, NLP-7, NLP-21 and UNC-1 encoding proteins^41^, localised with fluorescence reporters. Neuronal and AFD neuronal marker proteins are known to include some membrane proteins that have high molecular weight and complicated structures. Some studies indicated that membrane proteins may resist digestion^45^, and we may need to optimize extraction conditions and digestion to identify membrane proteins. Increasing the number of worms for proteomic analysis may improve the identification rate. Further, miniaturising the total volume of sample preparation would help to reduce protein loss. Sample preparation methods based on solid-phase extraction (SPE), e.g., miniaturised filter-aided sample preparation^46^ and in-StageTip method^47^, could be suitable approaches. In these methods, all sample processing, including cell lysis, reductive alkylation, digestion with trypsin and elution of purified peptides can be accomplished in a single enclosed reaction chamber. By combining SPE with alkyne groups for sample processing, we can recover azide-labelled proteins in a single reaction chamber with minimal surface losses. A single-pot solid-phase-enhanced sample preparation might become a preferable approach to recover enriched peptides with high recovery efficiency^48^.

In conclusion, we performed neuronal subclass selective proteomic analyses against all neurons or AFD neuronal subclass and identified 4,412 proteins and 1,834 proteins, respectively (Figs. 3, 6). These analyses demonstrated the feasibility of neuronal subclass-selective proteomic analysis. The analyses can be extended to elucidate proteomic differences among neurons using subclass-selective BONCAT.

## Supplementary information

Supplementary Figure S1.

Supplementary Table S1.

Supplementary Table S2.

Supplementary Table S3.

Supplementary Table S4.

## Data Availability

The datasets generated and/or analyzed during the current study are available in the jPOST repository, JPST000836.
