# Supplementary material for: Neuronal subclass-selective proteomic analysis in Caenorhabditis elegans
Source: Sci Rep. 2020 Aug 13;10:13840. doi: 10.1038/s41598-020-70692-w (PMC7426821; doi:10.1038/s41598-020-70692-w)
Supplement: Supplementary file 1 — Supplementary Figure S1. [file 41598_2020_70692_MOESM1_ESM.pdf]

Title

Neuronal subclass-selective proteomic analysis in *Caenorhabditis elegans*

Authors

Shunsuke Aburaya, Yuji Yamauchi, Takashi Hashimoto, Hiroyoshi Minakuchi, Wataru Aoki, Mitsuyoshi Ueda

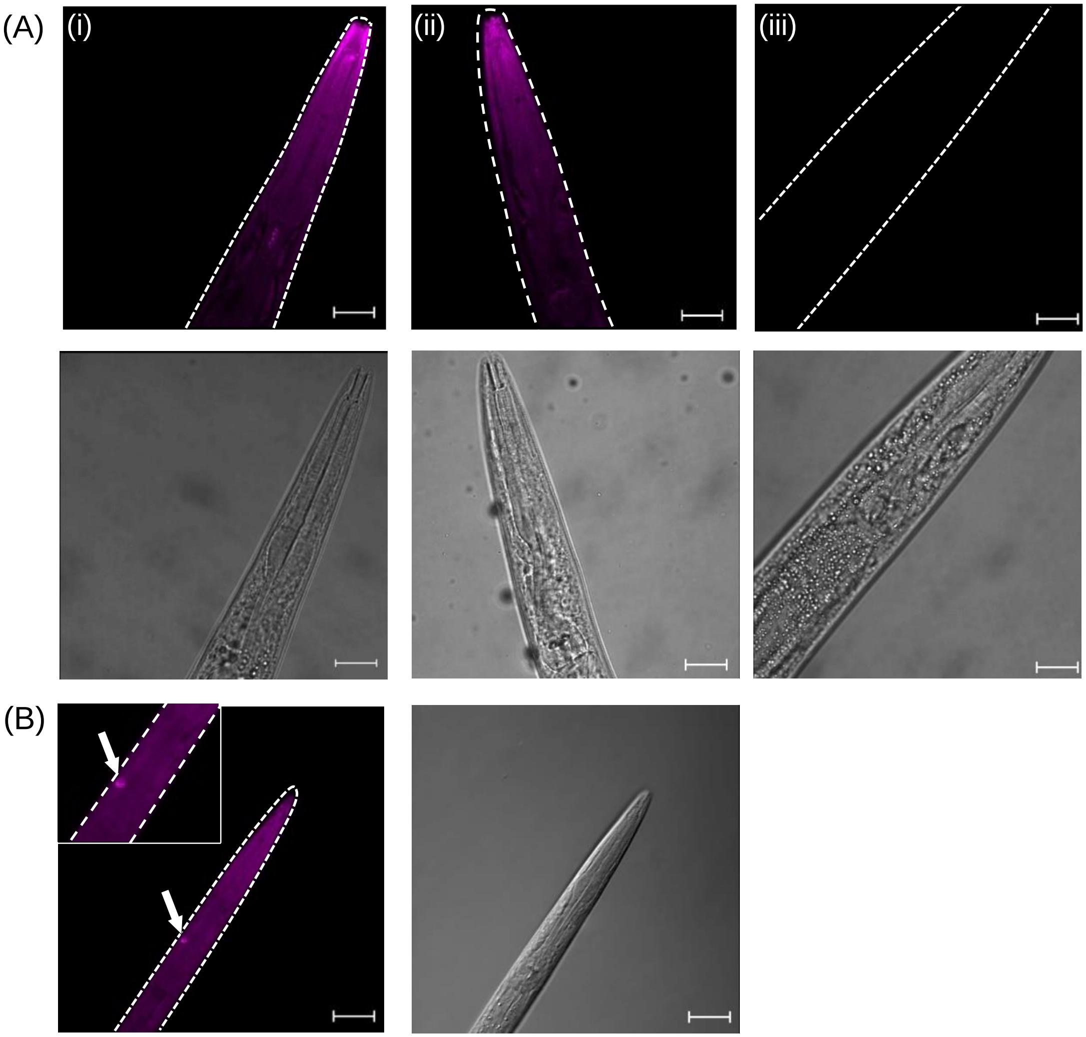

Supplementary Figure 1 TAMRA-DBCO staining of various *C. elegans* strains

(A) (i) Wild type N2 strain cultured with the Azf-labelled *E. coli* KY33 and stained with TAMRA-DBCO (ii) The SA1 strain cultured with the Phe-labeled *E. coli* KY33 and stained with TAMRA-DBCO (iii) The SA1 strain cultured with the Azf-labelled *E. coli* KY33 strain and not stained with TAMRA-DBCO. Scale bars indicate 20 μm.
(B) Confirmation of azide-phenylalanine incorporation at AFD neuronal cell in the SA2 strain cultured with the Azf-labeled E. coli KY33. Azide-proteins were stained with dibenzocyclooctyne-PEG4-Fluor 545 (TAMRA-DBCO). Scale bar indicates 20 μm. The arrow indicated the AFD neuronal cell.
